# Nonsurgical Periodontal Therapy Reduces Salivary and Gingival Crevicular Fluid YKL-40 and IL-6 Levels in Chronic Periodontitis

**DOI:** 10.3290/j.ohpd.a45086

**Published:** 2020-09-04

**Authors:** Zeynep Pinar Keles Yucel, Gonca Cayir Keles, Bahattin Avci, Burcu Ozkan Cetinkaya

**Affiliations:** a Assistant Professor, Giresun University, Faculty of Dentistry, Department of Periodontology, Giresun, Turkey. Designed the study, performed the clinical measurements, obtained the materials, drafted the manuscript, read and approved the final manuscript.; b Professor, Okan University, Faculty of Dentistry, Department of Periodontology, Istanbul, Turkey. Idea, supervised the study, proofread the manuscript, read and approved the final manuscript.; c Associate Professor, Ondokuzmayis University, Faculty of Medicine, Department of Biochemistry, Samsun, Turkey. Performed the biochemical analysis.; d Professor, Ondokuzmayis University, Faculty of Dentistry, Department of Periodontology, Samsun, Turkey. Performed statistical evaluation, contributed the discussion, read and approved the final manuscript.

**Keywords:** after-treatment, gingival crevicular fluid, human, interleukin-6, saliva, YKL-40 protein

## Abstract

**Purpose:**

A novel acute-phase protein, YKL-40, is known as an inflammation-associated glycoprotein. YKL-40 is shown to be linked to inflammation, endothelial dysfunction and tissue remodeling secreted by various cells and is also considered to be stimulated by cytokines such as interleukin-6 (IL-6). The present study aimed to investigate YKL-40 and IL-6 levels in saliva and gingival crevicular fluid (GCF) of patients with chronic periodontitis (CP) after non-surgical periodontal therapy for the first time.

**Materials and Methods:**

Twenty-six CP patients and 26 periodontally healthy individuals were enrolled. Clinical measurements were recorded; saliva and GCF samples were obtained at baseline and 1 and 3 months after non-surgical periodontal therapy. Levels of YKL-40 and IL-6 in saliva and GCF were analysed by ELISA.

**Results:**

Salivary and GCF YKL-40 and IL-6 levels were found to be statistically significantly higher in CP patients compared to healthy controls at baseline (p < 0.001). At 1 and 3 months after the completion of treatment, both YKL-40 and IL-6 levels in saliva and GCF had statistically significantly decreased compared with baseline values in CP patients (p < 0.001). On the other hand, no statistically significant difference was observed between 1 and 3 months in terms of salivary and GCF YKL-40 and IL-6 levels or any of the clinical findings (p > 0.016).

**Conclusion:**

Salivary and GCF YKL-40 levels may be useful to evaluate resolution of periodontal inflammation. Within the limits of this study, YKL-40 acute-phase protein might be a potential biomarker for detection of periodontitis and monitoring the response to periodontal therapy.

Periodontitis is a chronic inflammatory disease caused by periodontopathogens and characterised by irreversible destruction of tooth-supporting hard and soft tissues. Although the primary aetiological agent is microbial dental plaque, the onset, progression and severity of disease depend on the interactions between plaque bacteria and host immune response.^3,6^ The immune-inflammatory system is activated by bacterial endotoxins, and a variety of host inflammatory mediators that cause tissue breakdown are released, such as acute-phase proteins and cytokines.^6,40^ Acute-phase proteins play a critical role in the innate immune response and are secreted by many cells. There is an increasing body of evidence showing that levels of various acute-phase proteins change with periodontal disease in body fluids.^8,37,38^

YKL-40, a novel inflammatory glycoprotein, is an acute-phase protein with a molecular weight of 40 kDa and belongs to the chitinase protein family without enzymatic activity. YKL-40, also known as chitinase-3-like protein 1, is closely related to both acute and chronic inflammation, angiogenesis, endothelial dysfunction, and tissue remodeling. It is expressed by different types of cells including neutrophils, activated macrophages, differentiated vascular smooth muscle cells, chondrocytes, fibroblast-like synovial cells and cancer cells.^17,36^ Even though the biological functions of YKL-40 have not yet been fully elucidated, it promotes chemotaxis, cell attachment, cell differentiation, and migration, suggesting that this acute-phase protein has a pivotal role in host immune-inflammatory response.^36^ A growing body of clinical evidence reveals that circulation levels of YKL-40 are found to be elevated associated with the pathogenesis of various chronic inflammatory diseases, such as rheumatoid arthritis, osteoarthritis, atherosclerosis, diabetes mellitus, and cardiovascular disease.^17,19,32,35,36^ There are only a few studies investigating the relationship between YKL-40 and periodontal diseases, and they indicated an association between YKL-40 levels and periodontal diseases in gingival crevicular fluid (GCF)^9,21,22^ and serum.^21^ Therefore, YKL-40, known not to be a disease-specific molecule, seems to be a potential mediator in inflammatory diseases.^17^

The changes in YKL-40 secretion or production during inflammation demonstrate that this glycoprotein may be linked to a part of the cytokine network, which consists of soluble molecules triggering intracellular signaling pathways. It is well known that cytokines play a crucial role in acute-phase response and that they stimulate the synthesis of acute-phase proteins.^28,34^ Interleukin-6 (IL-6), a multifunctional cytokine, is the major regulator of acute-phase protein synthesis.^6,29^ Hence, IL-6 might stimulate the expression of YKL-40. It is also one of the most widely studied inflammatory markers in periodontal disease, with higher levels in the presence of periodontal diseases.^29^

The progression of chronic periodontitis (CP) can be managed by non-surgical periodontal therapy, and a balance between the host inflammatory mediators and immune response can show the efficacy of periodontal therapy in periodontitis patients.^27^ It has been proven that YKL-40 levels have both diagnostic and prognostic value for certain diseases.^23,26^

GCF is an essential material that contains many molecules and reflects the inflammatory events of periodontal tissues.^4^ Saliva is an important biological fluid containing a variety of locally and systemically derived products. Furthermore, saliva is readily accessible and can be collected in a noninvasive manner. It has become an emerging tool to evaluate oral and systemic health in recent years, because saliva analyses can identify various inflammation and tissue-destroying mediators. Thus, this fluid may be a significant source to identify inflammatory biomarkers of CP.^24,33^ The study’s hypothesis was that salivary YKL-40 levels and GCF in patients with CP might be elevated, but also decrease after nonsurgical periodontal therapy, considering that it can be stimulated by IL-6. To the authors’ knowledge, this is the first study aimed to comparatively evaluate salivary and GCF levels of YKL-40 and IL-6 before and after nonsurgical periodontal therapy in CP patients. The second objective was to explore any correlation of YKL-40 with IL-6 levels and clinical parameters. These data may provide information about the role of YKL-40 in the progression of periodontitis.

## Materials and Methods

### Study Population

A total of 52 participants (26 females and 26 males aged 26 to 49 years; mean age: 36.25 ± 5.14 years) admitted to the Department of Periodontology, Faculty of Dentistry, Adnan Menderes University, Aydin, Turkey from 2016 September to 2017 April were enrolled in this longitudinal study. Two groups were formed: 26 systemically and periodontally healthy individuals (H) (13 males and 13 females; mean age: 35.19 ± 5.21, age 26 to 46 years), and 26 systemically healthy generalised CP patients (13 males and 13 females; mean age: 37.31 ± 4.94, age 28 to 49 years). Participants were chosen to have a similar age range and gender distribution to prevent the influence of age and sex on clinical and biochemical findings. The study protocol and consent forms were approved by the Clinical Research Ethics Committee of the Faculty of Medicine, Adnan Menderes University (protocol number: 2016/1003). All individuals who agreed to participate voluntarily signed an informed consent in accordance with the Helsinki Declaration after receiving information about the study. This study was registered at ClinicalTrials.gov (NCT03507868).

The exclusion criteria were 1) presence of any systemic disease, 2) smoking, 3) a history of periodontal treatment in the past 6 months, 4) pregnancy or lactation, 5) usage of antibiotic, anti-inflammatory or any other drugs that affect periodontal status in the past 6 months.

All participants having 20 teeth or more (except third molars) underwent full-mouth periodontal clinical and radiographic examination. Clinical periodontal measurements include gingival index (GI),^25^ plaque index (PI),^39^ probing pocket depth (PPD), clinical attachment level (CAL) and bleeding on probing (BOP).^2^ Inclusion criteria for individuals were set based on the criteria proposed by the 1999 International World Workshop for a Classification of Periodontal Diseases and Conditions.^5^ The healthy control group consisted of individuals with PPD ≤ 3 mm without any clinical attachment loss or bone resorption at all sites. They exhibited no bleeding on probing and no sign of inflammation (GI = 0) in the entire mouth. The CP group had ≥ 4 teeth with PPD ≥ 5 mm and CAL ≥ 5 mm in each jaw and radiographic bone loss affecting >30% of the teeth. They had also GI ≥ 2 and bleeding scores >80% in the whole mouth.

The clinical measurements were assessed at six areas around each tooth: mesiobuccal, distobuccal, midbuccal, mesiolingual, distolingual, and midlingual. All clinical measurements were performed by a calibrated examiner (ZPKY) using a manual periodontal probe (Williams probe, Hu-Friedy; Chicago, IL, USA). Prior to actual measurement, 10 subjects were randomly selected for examiner calibration. The examiner evaluated the subjects on 2 separate occasions, 48 h apart. Calibration of the investigator was accepted if measurements at baseline and 48 h were >90% similar at the millimeter level.

### Collection of Samples

Saliva and GCF samples were collected one day after the clinical periodontal measurements (to avoid blood contamination) in the morning following an overnight fast. For saliva sampling, each participant was requested to not to eat or drink anything or chew gum. Whole saliva samples were obtained using the unstimulated saliva collection procedure. Participants expectorated into polypropylene tubes. The samples obtained were then centrifuged to remove cellular debris (10,000 x g for 10 min). The supernatants were immediately frozen at -80°C until analysis.

For GCF sampling, the buccal sides of mesial or distal interproximal sites of a single-rooted tooth were used. In the healthy control group, GCF samples were collected from each individual from a site of PPD ≤ 3 mm with no clinical signs of inflammation and no alveolar bone loss or BOP. For the CP group, one area from a tooth showing PPD > 5 mm with highest clinical signs of both inflammation and radiographic bone loss was selected for GCF sampling. Supragingival plaque was removed using a sterile curette without touching the gingival margins and the area was gently dried. Then, the area was isolated using cotton rolls to prevent saliva contamination. GCF was collected with a filter paper (Periopaper, Proflow; Amityville, NY, USA). Paper strips were gently placed into the gingival sulcus until met with mild resistance and left there for 30 s.^12^ Strips that were contaminated with blood and saliva were discarded. Then, GCF volume of per strip was measured by a precalibrated electronic impedance device (Periotron 8000, ProFlow; Amityville, NY, USA), whereupon it was immediately transferred into sterile polypropylene tubes and stored at -80°C until analysis.

### Nonsurgical Periodontal Therapy

CP patients received non-surgical periodontal therapy by the same researcher (ZPKY) after baseline saliva and GCF sampling. Non-surgical periodontal therapy included motivation, oral hygiene education, and scaling and root planing (SRP) procedures. SRP was performed using ultrasonic devices and manual scalers and specific curettes on a quadrant-by-quadrant basis once a week for four weeks. Every visit took 45 to 60 min. Root surfaces were instrumented under local anaesthesia if necessary. No antibiotics or any medications were applied in the treatment. Oral hygiene instructions including the modified Bass brushing technique and interdental cleaning procedures (dental floss or interproximal brushes based on patient’s requirement) were given to individuals. Motivation and the hygiene education were repeated at each visit during the treatment period. After completion of therapy, clinical periodontal measurements were repeated, and saliva and GCF samples were taken at 1 and 3 months in CP patients. GCF samples were obtained from the same site as prior to treatment.

### Analysis of YKL-40 and IL-6 Levels

One hundred twenty-five (125) µl of phosphate buffered saline (pH 7.4) was added to each Eppendorf centrifuge tube containing the strip. All tubes were vortexed for 1 min and then centrifuged at 10,000 x g for 5 min.

Saliva and GCF YKL-40 (Human YKL-40/CHI3L1 kit, Sunred Biological Technology; Shanghai, China) and IL-6 (Human IL-6 kit, Sunred Biological Technology) levels were measured using commercial ELISA kits. Analyses were performed according to the instructions provided by the manufacturer. The concentrations of YKL-40 (in 40 µl samples) and IL-6 (in 40 µl samples) were then determined by comparing the optical density of the samples to the standard curve.

Assay ranges of YKL-40 and IL-6 were 2 ng/ml to 300 ng/ ml and 3 pg/ml to 600 pg/ml, respectively. The sensitivities of YKL-40 and IL-6 were 1.115 ng/ml and 2.112 pg/ml, respectively. The amounts of YKL-40 and IL-6 in each sample were calculated based on dilutions, and results were expressed as total proteins in the 30-s GCF sample.

### Statistical Analysis

Power analysis was performed. Sixteen participants were required in each group to achieve 80% power and statistical significance at p < 0.05. It was decided to include 26 participants per group in order to increase the power of the study.

The Shapiro-Wilk test was used to investigate normality of distribution. The Mann-Whitney U-test was used for intergroup comparisons of all clinical and biochemical parameters as well as age. To compare variables between baseline and post-treatment at 1 and 3 months in the CP group, the Friedman non-parametric test was employed, followed by the Wilcoxon test if there was a statistically significant difference. The sex ratio between groups was evaluated with the chi-squared analysis. The relationship among salivary and GCF YKL-40 and IL-6 levels and clinical periodontal parameters was determined by the Spearman rank-correlation test. All statistical analyses were performed using a commercially available software program (SPSS Version 19.0; Chicago, IL, USA) with statistical significance set at p < 0.05.

Receiver operating characteristic (ROC) analysis was performed to quantify the diagnostic accuracy of YKL-40 and IL-6.

## Results

### Demographic Characteristics and Clinical Findings

Age and gender distribution showed no statistically significant difference between groups (p > 0.05). Full-mouth and sampling-site clinical periodontal findings are presented in Table 1. Both full-mouth and sampling-site clinical periodontal values were statistically significantly higher in CP patients compared to the healthy group (p < 0.001). All clinical periodontal values had decreased statistically significantly at 1 and 3 months after non-surgical periodontal therapy in CP patients (p < 0.001). PPD > 6 mm was found in 17.9% of sites at baseline, whereas no site was observed with PPD > 6 mm 1 and 3 months post-treatment. Nevertheless, periodontal parameters did not differ statistically significantly between 1 and 3 months after therapy, except for full-mouth BOP values (p > 0.016). Full-mouth BOP scores were statistically significantly lower at 3 months than at 1 month post-treatment (p < 0.016).

**Table 1:** Full-mouth and sampled site clinical parameters of healthy and CP groups (baseline, 1 and 3 months)

	Clinical parameter	Healthy	Chronic periodontitis		
			Baseline	1 month	3 months
Full-mouth periodontal evaluation	PPD (mm)	1.70 ± 0.27* 1.67 (1.33–2.39)	4.65 ± 0.32 4.57 (4.10–5.40)	2.93 ± 0.34* 2.88 (2.21–3.71)	2.87 ± 0.32* 2.81 (2.34–3.63)
	CAL (mm)	1.70 ± 0.27* 1.67 (1.33–2.39)	5.55 ± 0.57 5.51 (4.60–6.93)	4.06 ± 0.64* 3.93 (3.18–5.45)	4.13 ± 0.67* 3.93 (3.19–5.55)
	GI	0.19 ± 0.16* 0.20 (0.00–0.51)	2.61 ± 0.22 2.65 (2.03–2.89)	0.55 ± 0.25* 0.52 (0.11–1.03)	0.44 ± 0.20* 0.41 (0.16–0.92)
	PI	0.44 ± 0.19* 0.47 (0.09–0.87)	2.01 ± 0.43 1.98 (1.21–2.78)	0.89 ± 0.25* 0.84 (0.31–1.42)	0.77 ± 0.27* 0.75 (0.38–1.61)
	BOP (%)	0.52 ± 0.81* 0.00 (0.00–2.97)	88.96 ± 6.06 88.86 (75.00–98.71)	11.41 ± 5.13* 9.43 (5.33–25.64)	8.26 ± 4.01*§ 6.80 (4.48–19.87)
Sampled site periodontal evaluation	PPD (mm)	1.69 ± 0.47* 2.00 (1.00–2.00)	7.46 ± 0.51 7.00 (7.00–8.00)	3.57 ± 0.50* 4.00 (3.00–4.00)	3.46 ± 0.51* 3.00 (3.00–4.00)
	CAL (mm)	1.69 ± 0.47* 2.00 (1.00–2.00)	8.23 ± 0.65 8.00 (7.00–10.00)	6.15 ± 0.61* 6.00 (5.00–7.00)	6.23 ± 0.58* 6.00 (5.00–7.00)
	GI	0.00 ± 0.00* 0.00 (0.00–0.00)	2.76 ± 0.42 3.00 (2.00–3.00)	0.23 ± 0.42* 0.00 (0.00–1.00)	0.11 ± 0.32* 0.00 (0.00–1.00)
	PI	0.15 ± 0.36* 0.00 (0.00–1.00)	2.26 ± 0.53 2.00 (1.00–3.00)	0.38 ± 0.49* 0.00 (0.00–1.00)	0.19 ± 0.40* 0.00 (0.00–1.00)
	BOP (%)	0.00 ± 0.00* 0.00 (0.00–0.00)	100.00 ± 0.00 100.00 (100.00–100.00)	0.00 ± 0.00* 0.00 (0.00–0.00)	0.00 ± 0.00* 0.00 (0.00–0.00)
	GCF volume (μl)	0.06 ± 0.03* 0.06 (0.01–0.13)	0.94 ± 0.14 0.91 (0.65–1.26)	0.16 ± 0.06* 0.16 (0.07–0.35)	0.14 ± 0.04* 0.13 (0.07–0.28)

Data are expressed as the mean ± SD and median (minumum–maximum). *Statistically significant difference from baseline (p < 0.001). §Statistically significant difference between 1 and 3 months (p < 0.016).

### Biochemical Findings

Table 2 shows the salivary, GCF YKL-40 and IL-6 levels of the study groups. GCF YKL-40 and IL-6 total amounts were statistically significantly higher in CP than in healthy controls (p < 0.001). CP patients also had significantly elevated salivary levels of YKL-40 and IL-6 vs healthy individuals (p < 0.001).

**Table 2:** GCF and saliva YKL-40 and IL-6 levels of healthy and CP groups (baseline, 1 and 3 months)

Parameter	Healthy	Chronic periodontitis		
		Baseline	1 month	3 months
GCF YKL-40 total amount (ng/sample)	11.55 ± 1.57* 11.00 (9.01–14.16)	17.70 ± 1.23 17.70 (15.66–20.06)	12.93 ± 1.90* 12.22 (10.77–16.78)	12.82 ± 1.18*§ 12.84 (10.08–15.29)
Saliva YKL-40 (ng/mL)	82.34 ± 28.49* 89.26 (19.61–133.84)	171.72 ± 23.47 169.08 (142.26–213.11)	118.10 ± 16.74* 119.87 (84.37–146.36)	119.44 ± 25.43*§ 117.05 (77.53–160.38)
GCF IL-6 total amount (pg/sample)	9.92 ± 3.46* 9.26 (5.66–17.74)	18.24 ± 1.58 18.59 (15.38–20.86)	13.28 ± 2.20* 13.39 (8.69–16.94)	12.86 ± 2.22*§ 13.07 (6.79–16.84)
Saliva IL-6 (pg/mL)	93.97 ± 32.59* 97.10 (30.85–148.52)	203.29 ± 17.80 202.55 (171.15–243.20)	135.88 ± 27.30* 130.53 (90.43–182.73)	123.69 ± 33.65*§ 110.88 (62.00–181.26)

Data are expressed as the mean ± standard deviation and median (minumum–maximum). *Statistically significant difference from baseline (p < 0.001). § No statistically significant difference between1 and 3 months (p >0.016).

In CP patients, the results indicated a statistically significant reduction in both salivary and GCF total amount of YKL-40 at 1 and 3 months after non-surgical periodontal therapy compared to baseline (p < 0.001). Similarly, saliva and total IL-6 amounts in GCF were statistically significantly lower 1 and 3 months after treatment compared to baseline levels (p < 0.001). However, there was no statistically significant difference between 1 and 3 months after therapy (p > 0.016).

### Correlations

The total amount of GCF YKL-40 was statistically significantly positively correlated with the total amount of GCF IL-6 in healthy controls (r = 0.397, p = 0.040) as well as in the CP group before treatment (r = 0.443, p = 0.023). However, no statistically significant correlation was detected between salivary YKL-40 and IL-6 levels for both study groups (p > 0.05). In the CP group, a statistically significant positive relationship was observed between sampling-site CAL and GCF YKL-40 level 1 month after therapy (r = 0.414, p = 0.036). As for the correlations of all groups, both biomarkers were positively correlated with each other and also with the clinical periodontal parameters (PPD, CAL, GI and BOP) (p < 0.001).

### Diagnostic Value Findings

**Fig 1:**
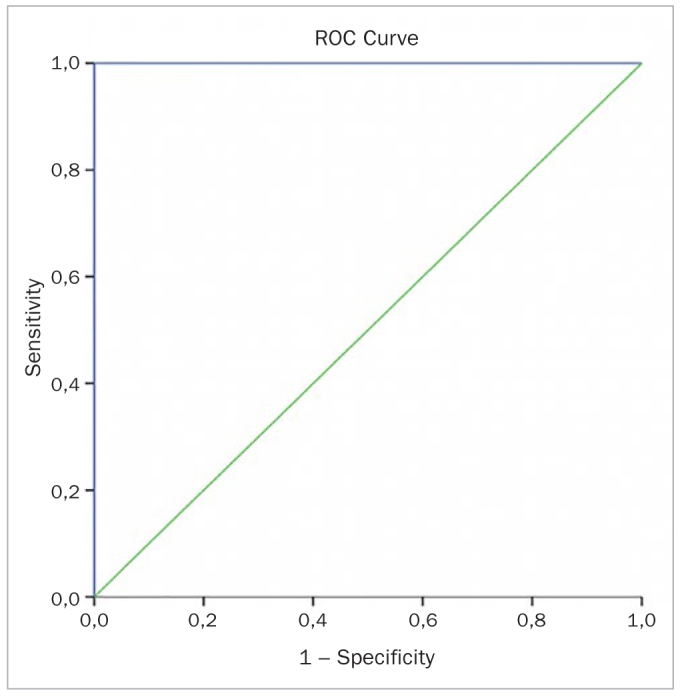
GCF YKL-4O ROC curve.

**Fig 2:**
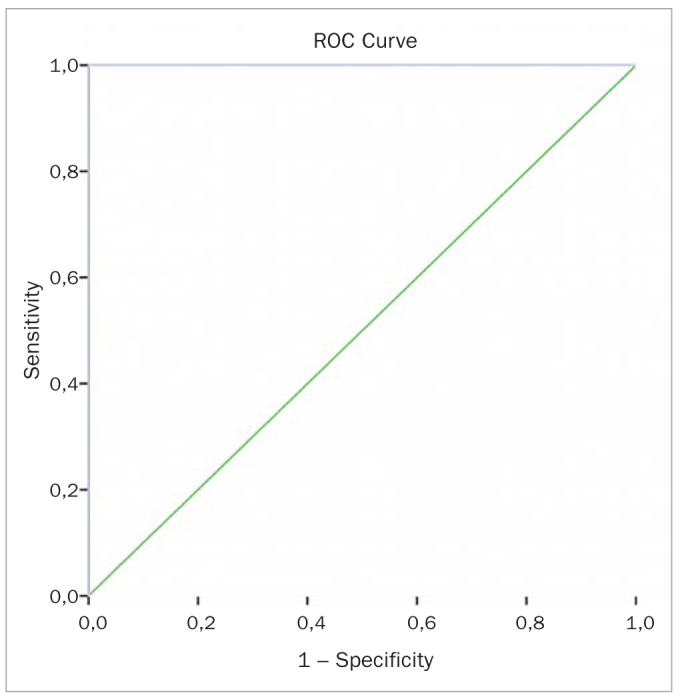
Saliva YKL-40 ROC curve.

**Fig 3:**
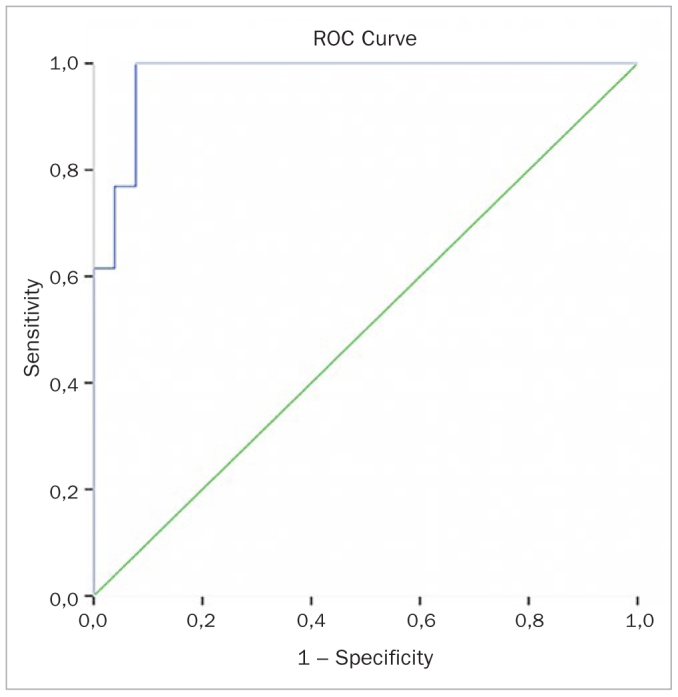
GCF IL-6 ROC curve.

**Fig 4:**
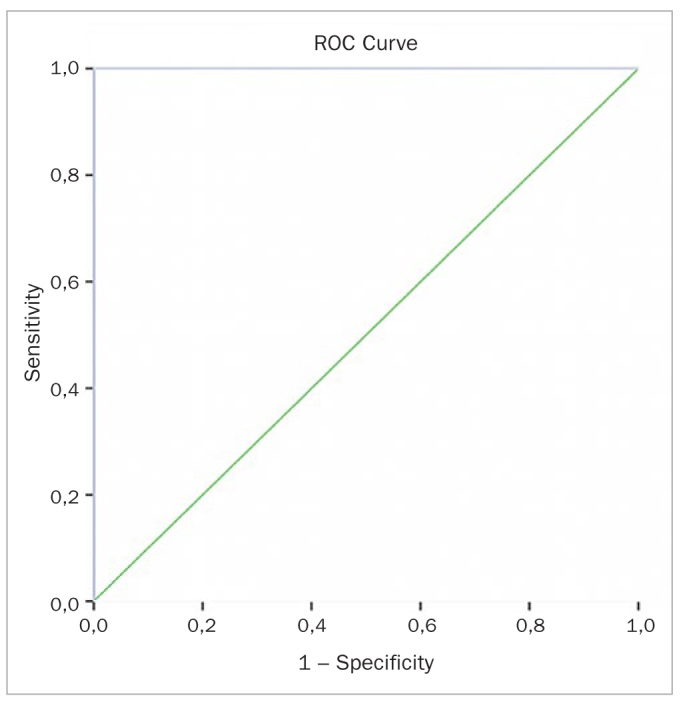
Saliva IL-6 ROC curve.

The potential use of YKL-40 and IL-6 as biomarkers to diagnose periodontal disease was analysed through ROC curves (Figs 1 to 4). Diagnostic values of YKL-40 and IL-6 both in saliva and GCF for chronic periodontitis are shown in Table 3.

**Table 3:** Diagnostic value of YKL-40 and IL-6 for CP

Marker	Cut-off	Sensitivity (%)	Specificity (%)	PPV (%)	NPV (%)	Accuracy (AUC, CI)
GCF YKL-40	14.91	100.00	100.00	100.00	100.00	1.000 (1.000–1.000)
Saliva YKL-40	138.05	100.00	100.00	100.00	100.00	.000 (1.000–1.000)
GCF IL-6	14.92	100.00	92.30	92.90	100.00	0.976 (0.940–1.000)
Saliva IL-6	159.84	100.00	100.00	100.00	100.00	1.000 (1.000–1.000)

AUC: area under the curve; CI: confidence interval; NPV: negative predictive value; PPV: positive predictive value.

## Discussion

The present study clearly showed that both the salivary and total amount of GCF YKL-40 increased in patients with CP compared to the control group and that it also statistically significantly decreased at 1 and 3 months following non-surgical periodontal therapy. Additionally, in all groups, GCF YKL-40 levels were statistically significantly correlated with periodontal clinical measurements as well as GCF IL-6 levels. In the literature, YKL-40 has been described as a new inflammatory marker of acute and chronic inflammation as well as cancer.^36^ YKL-40 levels have been shown to be elevated in a variety of inflammatory conditions, such as cardiovascular diseases, diabetes, and rheumatoid arthritis.^1,14,17,20,26,36,41^ There is evidence that serum and synovial fluid levels of glycoprotein were elevated in patients with rheumatoid arthritis.^19,20,31,41^ It was also shown that YKL-40 levels statistically significantly decreased when arthritis was treated.^41^ YKL-40 is thus thought to be a novel indicator of disease activity and poor prognosis in rheumatoid arthritis patients.^20,15,16,31,41^ One recent study found decreased levels of YKL-40 after treatment in patients with atrial fibrillation, and glycoprotein was considered to be a potential biomarker for predicting recurrence.^26^ Moreover, YKL-40 is released locally at the site of inflammatory reaction by activated macrophages and neutrophils. These acute-phase protein levels can increase during inflammation and decrease more rapidly after treatment because of local production.^36^ Therefore, YKL-40 may serve as a specific marker of inflammation for certain diseases to show disease activity and disease progression.

The most common outcome measures for defining the success of periodontal treatment are reduction of probing pocket depths, maintenance or increase of the clinical attachment levels and reduction of bleeding scores. As such, we have used these criteria to determine the success of periodontal therapy.^13^ On the other hand, disease progression or response to periodontal therapy is a result of the activated host inflammatory response.^30^ Accordingly, today’s diagnostic approaches involving clinical measurements of PPD, CAL, BOP, PI, and GI as well as radiographic findings are not sufficient to detect current disease activity. Therefore, it is very important to identify a reliable biomarker for early detection of periodontitis and defining progression. Rational diagnosis would also provide better management of periodontal disease. Not only does periodontitis appear locally, but it is also a risk factor for future systemic inflammatory diseases such as cardiovascular disease.^7^ Hence, the change of YKL-40 levels in body fluids before and after periodontal therapy may be essential to show the current state of periodontitis and also the future risk of systemic vascular damage. Additionally, its expression increases during the differentiation of monocytes to osteoclasts; therefore, increased production of this acute-phase protein may indicate bone destruction, which is also a characteristic of periodontitis.^10^

We preferred absorbent filter paper strips to obtain GCF samples instead of micropipettes or capillary tubing. Although all techniques have disadvantages, the micropipette technique makes it rather difficult to acquire adequate amounts of GCF, especially from periodontally healthy participants.^12^ Saliva, easily obtained without requiring any special equipment, is also an important material that can reflect all regions of mouth and contains immune-inflammatory cells and tissue products as well as GCF.^18^ The mediators were evaluated both in saliva and GCF in this study. Levels of YKL-40 and IL-6 in GCF were presented as the total amount of GCF in this study. An increasing body of evidence exists which shows that total amounts of cytokines or inflammatory mediators are more effective for identifying periodontal diseases and disease progression.^42^

Regarding YKL-40 acute-phase protein, despite abundant data on systemic diseases such as rheumatoid arthritis, data on periodontal diseases is quite limited. As expected, salivary and total GCF YKL-40 levels were statistically significantly higher in CP and decreased at 1 and 3 months after non-surgical periodontal therapy in the present study. These findings were in accordance with our previous report that demonstrated elevated GCF and serum levels of YKL-40 in both gingivitis and CP patients.^21^ A recent study investigating GCF YKL-40 levels in CP and diabetes patients also showed that the total amount of GCF YKL-40 was elevated both in diabetic and non-diabetic periodontitis patients and statistically significantly correlated with probing depth and GI scores.^22^ Likewise, CP patients with and without diabetes had reduced GCF YKL-40 levels after 6 weeks of SRP in another recent study.^9^ Our findings were also in agreement with those of the previous studies, despite methodological differences in terms of GCF collection. On the other hand, the present study also showed that reduced YKL-40 in both GCF and saliva after 1 month of SRP was maintained 3 months after therapy. Additionally, it is important to note that this study presented the first data about salivary levels of YKL-40 in periodontal disease. Recent researches have shown that salivary acute-phase proteins and cytokines were closely related to periodontal diseases.^11,38^ Supporting those findings, salivary YKL-40 levels were higher at baseline and decreased at 1 and 3 months after non-surgical periodontal therapy in patients with CP, as with GCF in our study. The present findings suggest that assessment of YKL-40 levels in saliva may be useful for diagnosis and screening of CP. Although saliva is not as specific as GCF, it seems to be sufficiently reliable to reflect the disease activity.

With regard to IL-6, it was not surprising that increased levels of the cytokine in CP at baseline and a marked decrease after therapy were observed. It has also been shown by previous studies.^6,37^ Thus, the present findings verified periodontal treatment success and resolution of inflammation. Evidence in the literature suggests that IL-6 may stimulate the release of YKL-40.^36^ Considering the results of the present study, it seems likely that YKL-40 levels in saliva and GCF may be regulated by IL-6. Particularly, the significant correlation between GCF levels of YKL-40 and IL-6 found here strengthen this view.

Our results clearly demonstrated that non-surgical periodontal therapy induced significant changes in salivary and GCF YKL-40 levels, as well as in IL-6, parallel to clinical periodontal healing. A statistically significant increase of salivary and GCF YKL-40 acute-phase protein in the presence of CP and the reduction following non-surgical periodontal therapy indicate that this inflammatory molecule might be secreted locally by periodontal tissues and could be closely associated with CP pathogenesis. However, there was no statistically significant difference between 1- and 3-month levels of YKL-40 and IL-6 in saliva and GCF. It is also important to consider that the statistically significant clinical improvements 1 month after periodontal therapy remained unchanged at the 3-month follow-up. This may be because there was no further decrease in PI scores or PPD and CAL at the 3-month follow-up compared to the 1-month results. Therefore, the clinical findings coincided with biochemical findings at follow-ups.

Assessing correlations, there was a statistically significant correlation between the total amount of YKL-40 and IL-6 in both the CP and healthy groups. The positive relationship between sample-site CAL and total amount of YKL-40 after therapy is one of the important findings of this study. On the other hand, considering all groups, GCF YKL-40 levels were positively correlated not only with GCF IL-6 levels but also sample-site PPD, CAL and GI. Similarly, Kido et al^22^ also showed a positive correlation between GCF YKL-40 and sample-site probing depth and GI in periodontitis. These limited but consistent findings also suggested that YKL-40 might be an effective acute-phase protein in CP. Moreover, diagnostic values of YKL-40 and IL-6 were examined in this study. The usefulness of YKL-40 and IL-6 as biomarkers of periodontal disease was verified through the analysis of their sensitivity and specificity using ROC curves. The area under the curve for YKL-40 in both saliva and GCF was 1.000. These values for IL-6 in saliva and GCF were 1.000 and 0.976, respectively. Considering the high accuracy, these results support the usefulness of YKL-40 and IL-6 as potential salivary and GCF diagnostic biomarkers.

## Conclusion

Non-surgical periodontal treatment appears to substantially reduce YKL-40 acute-phase protein levels in chronic periodontitis. Evaluating the levels of this protein in saliva and GCF might help monitor the response to periodontal therapy and the progression of periodontal inflammation. Thus, the level of YKL-40 in saliva and GCF might be a valuable means of identifying the current state of periodontitis. Further studies investigating YKL-40 levels in various biofluids combined with microbiological analysis are needed to confirm these findings and better understand the mechanism of this protein.
